# Spontaneous Participation in Secondary Prevention Programs: The Role of Psychosocial Predictors

**DOI:** 10.3390/ijerph17176298

**Published:** 2020-08-29

**Authors:** Alessandra Gorini, Mattia Giuliani, Giulia Marton, Laura Vergani, Simone Barbieri, Fabrizio Veglia, Elena Tremoli

**Affiliations:** 1 Department of Oncology and Hemato-Oncology, University of Milan, 20122 Milan, Italy; giulia.marton@ieo.it (G.M.); laura.vergani@ieo.it (L.V.); 2 Applied Research Division for Cognitive and Psychological Science, European Institute of Oncology IRCCS, 20141 Milan, Italy; 3 IRCCS Centro Cardiologico Monzino, 20138 Milan, Italy; mattia.giuliani@ccfm.it (M.G.); simone.barbieri@ccfm.it (S.B.); fabrizio.veglia@ccfm.it (F.V.); elena.tremoli@ccfm.it (E.T.)

**Keywords:** spontaneous participation in secondary prevention, health psychology, psychosocial predictors, Facebook sampling, non-communicable diseases (NCDs)

## Abstract

Disease prevention is a multifaceted construct that has been widely studied. Nevertheless, in spite of its importance, it is still not sufficiently considered by the general population. Since the reasons for this lack of consideration are not yet fully understood, we created an Online Prevention Survey (OPS) to investigate the role of both sociodemographic and psychological factors in predicting individuals’ spontaneous participation in secondary prevention programs. The results revealed that younger people, men, manual workers, unemployed people, and those who do not regularly practise physical activity were less likely to spontaneously participate in such programs. Furthermore, an analysis of the psychological determinants of the willingness to participate in secondary prevention programs showed that depressive symptoms negatively predict it, while an individual’s perception of receiving high social support acts as a positive predictor. Based on these results, we suggest the need for implementing new tailored approaches to promote prevention initiatives to those segments of the population which are more reluctant to spontaneously undertake prevention paths.

## 1. Introduction

Disease prevention is an umbrella term, including activities, actions, and interventions aimed to promote and preserve individuals’ health to decrease the incidence of disease outcomes in the population. Broadly, disease prevention can be divided into three main categories: primary, secondary, and tertiary prevention. The present study is specifically focused on secondary prevention, which includes preventive measures that lead to early diagnosis and prompt treatment of a disease, illness, or injury to prevent the development of severe problems. Secondary prevention primarily comprises periodical screening initiatives (i.e., blood pressure controls, physical examinations, dental exams, and so on), crucial to reducing the huge number of preventable deaths that occur every year worldwide, most of which are caused by non-communicable diseases (NCDs) [1].

NCDs are non-infectious health conditions originating from a combination of genetic, physiological, environmental, and behavioural factors that cannot be transmitted from person to person. Recent data from the World Health Organization (WHO) have shown that NCDs kill 41 million people every year, responsible for 71% of all deaths globally. Among them, cardiovascular diseases (CVD) account for the most deaths (17.9 million people annually), followed by cancer that causes around 9.0 million deaths per year [2]. In 2011 and 2013, both the United Nations Assembly and the WHO recognized the importance of developing new preventive politics to reduce the damage caused by NCDs [3]. However, the effectiveness of prevention programs is deeply dependent on the population participation rate, which is still extremely low [4].

In the last decade, researchers have attempted to identify factors related to poor participation in prevention initiatives and, in particular, the limited adherence to secondary prevention programs, finding that specific sociodemographic factors may have a significant role in health-related decisions. Specifically, it was found that age and gender (i.e., participation in prevention is higher in older patients and women), marital status (i.e., men with a spouse are more likely to participate in screening programs) [5,6,7], a high family income, health insurance coverage and/or a usual source of care, and living in an urban area make people more prone to participate in preventive health checks [8]. Furthermore, positive lifestyle factors, such as being a non-smoker and regular physical activity, were also found to be associated with a higher participation rate in secondary prevention programs [7]. Conversely, among the reasons given by those who do not participate in secondary prevention programs, Wall and Teeland showed that the most common was the lack of time or hindrances at work as well as the feeling of being quite healthy at present [7]. Lakerveld and colleagues also highlighted the role of other practical aspects, such as costs, time investment, and distance from medical centres as common causes for not starting or abandoning secondary preventive health initiatives [9].

Despite the vast literature on factors involved in secondary prevention participation, very few studies have explored the impact of psychological factors in prevention-related decisions. With regard to cancer prevention, for example, available results show that depressive and anxiety symptoms [6,10,11,12], loneliness, social exclusion, stress, pessimism, embarrassment and low self-esteem, low self-efficacy, lack of self-regulation capability, and low perceived autonomy [13] are negatively associated with the willingness to participate in prevention initiatives. Similarly, depression and anxiety have been found to decrease participation in secondary prevention programs, even for cardiovascular diseases [14,15,16].

Thus, given the lack of studies investigating the role of psychological factors in spontaneous prevention-related decisions, the main aim of the present study was to develop an online survey to investigate if depression, anxiety, perceived stress, perceived social support, general self-efficacy, and personality traits (i.e., negative affectivity and social inhibition), known to play a role in adherence to illness-specific prevention programs [6,10,11,12,17,18,19], could act as positive or negative predictors even in the spontaneous decision to participate in secondary prevention programs among the general population. This study also had two secondary aims: (a) to investigate the reasons due to which people choose to not participate in secondary prevention programs and (b) to analyse which type of secondary prevention programs are the most selected by the general population.

## 2. Materials and Methods

### 2.1. Participants

The original sample consisted of 1152 participants who completed the entire survey. These responders represented 68% of the total number of individuals who accessed the online questionnaire (32% did not complete it and were excluded from the study). The inclusion criteria for participation were: (a) being an Italian native speaker and (b) age between 18 and 75 years. Responders who said that their participation in secondary prevention programs was mandatory (for example, requested by their employers) or related to a pre-existing condition were excluded from the study. The final sample comprised 1049 subjects, 638 women (60.8%), and 411 men (39.2%).

### 2.2. The Online Prevention Survey (OPS)

The Online Prevention Survey (OPS) was developed by an Italian research team and consisted of ad hoc and validated questionnaires. To recruit participants, a purposive sampling technique without a pre-determined sampling frame, wherein the authors invited all of their Facebook contacts to complete the survey, was used. Participation was voluntary and free; no incentives were offered to complete the survey. Following the Helsinki declaration, no ethics committee authorization was needed. The survey was preceded by a short introduction explaining the general aim of the study, followed by four main sections specifically devoted to (a) investigate the responders’ secondary prevention behaviours, (b) collect their sociodemographic characteristics, (c) investigate the responders’ lifestyle habits (as part of their primary prevention behaviours), and (d) conduct a psychological self-assessment.

#### 2.2.1. Secondary Prevention Behaviours

In the first section of the OPS, the following question was asked: “In the last three years, have you ever participated in secondary prevention initiatives in the absence of specific clinical symptomatology or overt diseases, in a completely spontaneous way? (For example: cancer or cardiovascular screening, blood test, etc.)”. In case of a positive answer, information about the type and the frequency (e.g., “Only one time”, “Annual”, “Biannual”, or “Every three years”) of the prevention initiative(s) were asked. Conversely, in case of a negative answer, the reasons due to which subjects did not participate in any prevention screening were investigated. Participants’ answers were then categorized into the following categories: disregard (i.e., “I am not interested in doing prevention”), uneasiness (i.e., “I am not comfortable in doing some kind of exams”), fear of the outcome (i.e., “I am worried about the results” or “I do not want to know if I have a disease, because I would be afraid”), distrust in health care facilities (i.e., “I do not trust doctors”, “Hospitals do not think about my health, they only think about money”, or “I do not think that doctors could really help me”), logistic barriers (i.e., “I have no time” or “The hospital is really far from home and/or work”), health problems (i.e., “I am not independent because of my health, so I should depend on others” or “My health would not allow me to participate in prevention programs”), disinformation (i.e., “I never heard about health preventive programs”, “I thought that my general practitioner should have tell me to participate”, or “I thought that only people who have a disease should participate in those programs”), laziness (i.e., “It would be too tiring for me”), and other reasons (i.e., “My life is too complicated”). Based on the answers about their secondary prevention habits, the initial sample was divided into two subgroups: the first group included those subjects who did not participate in any secondary prevention program (Group NP, *n* = 277), while the second group included those who voluntarily participated in at least one secondary prevention program in the last three years (Group P, *n* = 772).

#### 2.2.2. Sociodemographic Assessment

In the second section of the survey, seven sociodemographic questions were administered to investigate the respondents’ age, gender, marital status, education, occupation, and if they had any offspring.

#### 2.2.3. Lifestyle Habits (as part of primary prevention behaviours)

In the third section of the OPS, seven questions investigated if respondents engaged in some primary prevention behaviours related to smoking habits, alcohol consumption, and physical activity.

#### 2.2.4. Psychological Assessment

The fourth section of the OPS comprised psychological self-assessment and included the evaluation of depressive symptoms (i.e., Beck Depression Inventory-II, BDI-II) [20], anxiety symptoms (i.e., State-Trait Anxiety Inventory Form Y, STAI-Y; Health Anxiety Questionnaire, HAQ) [21,22], level of personal distress (i.e., Perceived Stress Scale, PSS) [23], perceived social support (i.e., Multidimensional Scale of Perceived Social Support, MSPSS) [24], type “D” personality (i.e., Type-D Personality Scale, DS-14) [25], and self-efficacy (i.e., General Self-Efficacy Scale, GSE) [26].

##### Beck Depression Inventory-II (BDI-II)

The BDI-II is widely used to evaluate the severity of depressive symptoms in adult and adolescent patients. It consists of 21 items with four response options, ranging from 0 (i.e., “Not Present”) to 3 (i.e., “Severe”). It provides four categories of symptoms based on the obtained score: 0–13 (minimal depression), 14–19 (mild depression), 20–28 (moderate depression), and 29–63 (severe depression) [27].

##### State-Trait Anxiety Inventory Form Y (STAI-Y)

The STAI-Y is widely used to measure trait and state anxiety. Form Y is the most common version and comprises 20 items for assessing trait anxiety (e.g., “I worry too much over something that really doesn’t matter”) and 20 items for state anxiety (e.g., “I am tense”, “I am worried”, or “I feel calm”). All items are rated on a 4-point Likert scale, from “almost never” to “almost always”, and no cut-off points are used: the higher the total score, the more severe the anxiety symptoms.

##### Health Anxiety Questionnaire (HAQ)

The HAQ is a self-report questionnaire that comprises 21 items describing health anxiety-related symptoms. Each item is scored on a four-point Likert scale (i.e., from 0 to 3) describing the frequency of each symptom: 0 (i.e., not at all or rarely), 1 (i.e., sometimes), 2 (i.e., often), and 3 (i.e., most of the time).

##### Perceived Stress Scale (PSS)

The PSS is the most widely used self-report questionnaire for measuring distress perception. The items evaluate the frequency of feelings and thoughts related to distress perception during the last month. The scores range from 0 to 40, with higher scores indicating a higher level of perceived distress. The score interpretation is based upon three value categories: 0–13 (i.e., low stress), 14–26 (i.e., moderate stress), and 27–40 (i.e., high stress) [28].

##### Multidimensional Scale of Perceived Social Support (MSPSS)

The MSPSS is a 12-item questionnaire designed to measure perceptions of support from three sources: family, friends, and a significant other. The total score ranges from 1 to 7, with three interpretation categories: from 1 to 2.9 (i.e., the perception of low social support), from 3 to 5 (i.e., the perception of moderate social support), and from 5.1 to 7 (i.e., the perception of high social support) [29].

##### Type-D Personality Scale (DS-14)

The DS-14 is a brief self-report questionnaire, which is used worldwide to evaluate type-D personality traits: negative affectivity (NA) and social inhibition (SI). The DS-14 comprises 14 items, each evaluated on a scale between 0 (i.e., false) and 4 (i.e., true). It provides two separate scores for NA and SI, each in the range from 0 to 28. A cut-off score of ≥10 means the presence of a maladaptive personality trait [30].

##### General Self-Efficacy Scale (GSE)

The GSE is a 10-item questionnaire widely applied to assess optimistic self-beliefs (self-efficacy) used to cope with a variety of difficulties in life. The total score ranges from 10 to 40, with higher scores indicating higher perceived general self-efficacy and lower scores indicating lower perceived general self-efficacy. It has been proposed that a score ≤18 indicates a low level of perceived self-efficacy, while a score >18 indicates a normal level of perceived self-efficacy [31].

### 2.3. Statistical Analysis

All the analyses were performed using SAS version 9.4 (SAS Institute Inc, Cary, NC, USA). To evaluate both sociodemographic and psychological differences between groups, independent samples t-test (for normally distributed variables), Mann–Whitney U test (for not normally distributed variables), and χ² test (for categorical variables) were performed. Normally distributed variables included age, education, Perceived Stress Scale (PSS), General Self-Efficacy Scale (GSE), State-Trait Anxiety Inventory (Trait anxiety, STAI-T), and Multidimensional Scale of Perceived Social Support (MSPSS). Non-normal distributed variables included Beck Depression Inventory-II (BDI-II), Health Anxiety Questionnaire (HAQ), and Type-D Scale (DS-14). Categorical variables included sex, marital status, occupation, offspring, smoke, alcohol consumption, and physical activity. Though it was possible to consider the respondents’ answers to psychological questionnaires as categorical variables based on their available cut-off, since they were used as covariates, we considered them as continuous variables. 

Logistic LR forward stepwise regression was performed to investigate which variables could predict spontaneous participation in disease prevention programs. In our logistic model, the spontaneous participation in preventive programs in the last three years was considered as the dependent variable and was classified in the following way:

- 0 if the participant did not spontaneously participate in secondary prevention programs in the last three years (Group NP);
- 1 if the participant spontaneously participated in at least one secondary prevention program in the last three years (Group P).

The significance level chosen for each analysis was p < 0.05 (two-tailed).

## 3. Results

### 3.1. Sociodemographic Assessment

The entire OPS sample was primarily composed of middle-aged participants (mean = 54.38 years, SD = 10.80, range = 19–89) who had achieved compulsory Italian education (mean = 14.44 years, SD = 3.78). The majority of them were women (60.8%) and married (68%).

Respondents in Group P were slightly older than those included in Group NP (Group P: mean = 55.82 years, SD = 9.55, range = 25–78; Group NP: mean = 50.38 years; SD = 12.91; range = 19–89; *p* < 0.001). A detailed description of the sociodemographic characteristics of the two subsamples is reported in Table 1.

### 3.2. Primary Prevention Behaviours

We found significative differences between the two groups both in smoking behaviour and the frequency of physical activity. In particular, Group P included less smokers than Group NP (non-smokers in Group P: 86.4%; non-smokers in Group NP: 80.1%; *p* = 0.013) and were more accustomed to physical activity (Group P: 59.1%; Group NP: 53.4%; *p* < 0.001). All these data are reported in Table 1.

### 3.3. Secondary Prevention Behaviours 

Regarding secondary prevention behaviours, the majority of participants included in Group P declared that they used to participate in at least one secondary prevention initiative once a year (44.8%) and that this was usually related to oncological diseases (81.3%), while only 12.6% underwent cardiovascular prevention screenings. Finally, group NP rated both disregard (30.4%) and logistical barriers (30.8%), such as distance, travel, and time costs (Table 2), as the main reasons for not participating in prevention programs.

### 3.4. Psychological Assessment

Group P showed significantly lower level of depressive symptoms (Group P: median = 8, IQR = 4-13; Group NP: median = 10, IQR = 5–16; *p* < 0.001) and perceived stress (Group P: mean = 14.63, SD = 7.1; Group NP: mean = 16.82, SD = 8.5; *p* < 0.001), and higher general self-esteem (Group P: mean = 29.04, SD = 4.5; Group NP: mean = 28.35, SD = 5.2; *p* = 0.033) than Group NP. With regard to trait anxiety, Group P showed a significantly lower score than Group NP (Group P: mean = 38.92, SD = 11.2; Group NP: mean = 42.22, SD = 12.4; *p* < 0.001), whereas relative to health anxiety, Group P rated significantly lower in the interference subscale (Group P: median = 0, IQR = 0–2; Group NP: median = 1, IQR = 0–2.5; *p* = 0.039) and significantly higher in the reassurance subscale (Group P: median = 3, IQR = 2–4; Group NP: median = 2, IQR = 1–4; *p* = 0.024) compared with Group NP. Group P also showed significantly higher scores in perceived social support from family (Group P: mean = 5.17, SD = 1.3; Group NP: mean = 4.87, SD = 1.5; *p* = 0.002), friends (Group P: mean = 4.84, SD = 1.2; Group NP: mean = 4.43, SD = 1.4; *p* < 0.001), and significant other (Group P: mean = 5.51, SD = 1.2; Group NP: mean = 5.30, SD = 1.4; *p* = 0.016), and total score (Group P: mean = 5.17, SD = 1.1; Group NP: mean = 4.87, SD = 1.2; *p* < 0.001) compared with Group NP. Group P also reported both negative affectivity (Group P: median = 9, IQR = 4–15; Group NP: median = 11, IQR = 5–17; *p* = 0.001) and social inhibition sub-threshold scores (Group P: median = 7, IQR = 3–13; Group NP: median = 10, IQR = 3–15; *p* = 0.003), which significantly differed from Group NP’s scores. All these data are presented in Table 3.

### 3.5. Multivariate Logistic Regression

Multivariate logistic regression revealed that age, gender, occupation, depressive symptoms, and perceived social support could act as predictive factors in spontaneous participation in prevention programs. Specifically, older people seemed to be more prone to spontaneously participate in secondary prevention programs than younger people, as an increase of one year corresponded to an 8% increase in the odds of spontaneous participation in a prevention program (OR = 1.08, 95% CI = 1.06–1.09, *p* < 0.001).

Men seemed to be less motivated than women to spontaneously participate in secondary prevention programs (OR = 0.31, 95% CI = 0.22–0.43, *p* < 0.001), while subjects that regularly practised physical activity seemed to be more likely to participate in prevention programs (OR = 1.48, 95% CI = 1.08–2.02, *p* = 0.013) compared with those with more sedentary lifestyles. The multivariate logistic regression also highlighted the predictive effect of working activity on prevention habits indicating that compared with manager and practitioners, subjects with a service job seemed to have a greater likelihood of spontaneously participating in prevention programs (OR = 1.12, 95% CI = 0.77–1.64, *p* = 0.006), while subjects with manual jobs or unemployed subjects seemed to show a lesser likelihood (OR = 0.48, 95% CI = 0.29–0.79, *p* = 0.007) of doing so. Conversely, being retired did not significantly influence spontaneous participation in prevention programs.

Finally, subjects receiving limited social support (OR = 1.02, 95% CI = 1.01–1.04, *p* < 0.001) and those showing depressive symptoms (OR = 0.97, 95% CI = 0.96–0.99, *p* = 0.003) seemed to do less spontaneous prevention compared with others. All these results are reported in Table 4.

## 4. Discussion

According to the literature, the role of sociodemographic factors in the decision to not participate in secondary prevention programs is still contradictory. However, general trends emerging from secondary prevention health studies can be identified: non-participants are likely to be younger and male [8]. Consistent with this data, our results showed that older people tend to be more likely to engage in spontaneous participation in secondary prevention programs than younger people. We also found that sex seems to play an important role, as men showed a 69% lower probability in spontaneous adherence to secondary prevention initiatives compared with women [32,33,34,35].

Other than age and sex, even occupation could predict spontaneous participation in secondary prevention programs, as demonstrated by the fact that subjects who did service jobs seemed to be more likely to participate in secondary prevention programs than both managers and practitioners, whereas manual workers and unemployed people seemed to be less likely to do it. We also observed that subjects who regularly practised any form of primary prevention (i.e., those who practised physical activity and/or did not smoke) appeared to be more involved in secondary preventive initiatives compared with others. These data suggest the existence of a predictable association between the two forms of prevention. However, to the best of our knowledge, no studies have systematically examined the role of lifestyle habits and occupation in determining the willingness to participate in secondary prevention programs, and more studies are needed to understand such a relation.

Other than analysing the potential role of sociodemographic variables, this study was the first to assess the role of different psychological variables as possible predictive factors of spontaneous willingness to participate in secondary prevention initiatives, finding that both self-reported depressive symptoms and low self-perceived social support appear to be related to it. Specifically, we noticed an inverse relationship between the occurrence of depression symptoms and spontaneous participation in secondary prevention programs: the more severe the self-reported depressive symptoms, the lower the probability of spontaneous participation in secondary prevention programs. This observation is in line with the data reported by Pirraglia and colleagues, who highlighted that people suffering from depression or depressive mood are less prone to adhere to secondary prevention programs and, in particular, to cancer screening initiatives [12], as indicated by data from Myong and colleagues about colorectal cancer screening [5]. A possible interpretation of these findings is that depressive symptoms, including anhedonia, apathy, abulia, little investment in self-care, and the lack of future perspective could act as negative predictors in the decision to spontaneously take part in secondary prevention programs. 

Conversely, we found that self-perceived social support seems to act as a positive predictor in the spontaneous decision to participate in secondary prevention programs, in line with previous research, showing a positive association between social support and health-promoting behaviours, such as physical activity and adherence to medical prescriptions [36].

The second objective of this study was to explore the motivations of participants for not spontaneously taking part in secondary prevention programs. We found that the most common reported motivations were disregard (30.4%) and logistical hindrances (30.8%), followed by the fear of outcome (9.4%). These data are in line with those reported by the only other existing study that evaluated the willingness to participate in spontaneous prevention for cardiometabolic diseases [4], in which the barriers in health check were the fear of outcome and time investment, which, in our study, was included in the logistical hindrances category.

Finally, we wanted to assess which types of secondary prevention were performed in the last three years by the participants. To do this, we categorized the participants’ responses into three main clusters: cardiovascular prevention, oncological prevention, and other types of prevention (such as eye exams, blood tests, ENT exams, and dental exams) that were not self-excluding to each other (i.e., a participant who indicated both cardiovascular and oncological prevention was included in both cardiovascular prevention and oncological prevention categories). Data showed that only about 13% of participants underwent secondary cardiovascular prevention, whereas 81% participated in cancer screening initiatives, possibly because there are more organized and well-known screening programs for cancer (i.e., for breast, cervical, and colorectal cancer) than for other illnesses. Nevertheless, they appear particularly interesting from a cognitive point of view, and even counterintuitive, if we consider that cardiovascular diseases (CVDs) cause the highest number of deaths in the European Union [37] and worldwide [38].

Moreover, while men are wrongly considered to be more at risk than women regarding the incidence of CVDs, growing data on women’s unique cardiovascular risk factors and the need to tailor screening and treatment accordingly were recently highlighted [39]. These data strongly underline that there is still a significant lack of knowledge in the population regarding the impact of CVDs and consequently the important role of its prevention. This may be due, perhaps, to the fact that other diseases, such as cancer, have received specific governmental attention as well as dedicated resources at the national level that CVDs have not received [40].

### Strength and Limitations

The primary strengths of this study were as follows: (a) we analysed the effect of the psychological variables, and not only sociodemographic variables, on the willingness to participate in disease prevention initiatives, since they are crucial in any decision-making process, including those related to health; (b) we observed the preventive behaviours in the general population, instead of considering only specific clinical samples undergoing prevention; and (c) we conducted the study in Italy where, to our knowledge, there are no data about psychosocial determinants of prevention behaviours.

Regarding limitations, we cannot exclude a sample selection bias due to the Facebook-based recruitment. In particular, our sample’s mean age was about fifty-four years, which suggests that the online administration limited the participation of the elderly. Since older people may have specific attitudes toward spontaneous participation in health prevention programs, further studies are needed to analyse this issue. Moreover, since recruitment was performed inviting the authors’ Facebook contacts and asking them to share the survey with their contacts, we did not have specific information on how many individuals followed the Facebook page on which the survey was shared. This did not allow a comparison between individuals who followed the page and those who completed the survey. Nevertheless, several studies have demonstrated that Facebook-based recruitment successfully achieves representative samples of target populations [41], suggesting that our data can be considered reliable and replicable, at least for the medium age population. Among the social media platforms used for health studies, Facebook is the most used [42]. Another limitation of this study was the sole reliance on self-report measures, which could have led to an over- or under-estimation of the examined psychological characteristics of the sample. However, if this is truly the case, we have certainly opened the way to other studies, which should deepen the role of psychological characteristics in disease prevention.

## 5. Conclusions

Other than confirming the previous observations about the role of sociodemographic factors in health-related decisions, the present study highlights the role of psychological factors, such as depressive symptoms and perceived social support, as negative and positive predictors for the spontaneous decision to participate in secondary prevention programs. Moreover, our results also provide extremely interesting information regarding the disproportion between very widespread diseases and the limited participation of subjects in secondary spontaneous prevention measures that are necessary to reduce the impact of the disease in the general population. In particular, even though cardiovascular diseases are the leading cause of death globally, very few people decide to participate in specific heart-related prevention strategies. Finally, we observed that one of the main reasons that lead respondents to not participate in spontaneous secondary prevention is disregard, indicating the need for more effective preventive campaigns.

Taken together, these data suggest that it is fundamental to strengthen the actual communication messages concerning secondary prevention, creating tailored and personalized initiatives specific for different diseases that take into account various information about the individuals, including their psychosocial characteristics and cognitive frame involved in their health-related choices, to increase the general participation rate in secondary prevention programs [43,44,45]. Giving the right amount of importance to psychological health concerns is a crucial strategy not only in the field of prevention, but also in the management of existing acute and chronic illnesses to improve patients’ quality of life [46,47,48].

## Figures and Tables

**Table 1:** Sociodemographic characteristics and primary prevention behaviours.

Sociodemographic Variables	OPS		Group P		Group NP		Group P vs. Group NP *p*-Value
	***N***	**Mean (SD)**	***N***	**Mean (SD)**	***N***	**Mean (SD)**	
Age	973	54.38 (10.80)	716	55.82 (9.55)	257	50.38 (12.91)	**<0.001 ^1^**
Education	1049	14.44 (3.78)	772	14.49 (3.72)	277	14.30 (3.93)	0.473 ^1^
		*N (%)*	*N (%)*		*N (%)*		
Sex	Female	638 (60.8)	502 (65.0)		136 (49.1)		**<0.001 ^2^**
	Male	411 (39.2)	270 (35.0)		141 (50.9)		
Maritalstatus	Single	122 (11.6)	75 (9.7)		47 (17)		**<0.001 ^2^**
	Unmarried couples	98 (9.3)	62 (8)		36 (13)		
	Married couples	713 (68.0)	550 (71.2)		163 (58.8)		
	Divorced	93 (8.9)	65 (8.4)		28 (10.1)		
	Widowed	23 (2.2)	20 (2.6)		3 (1.1)		
Occupation	Manager or practitioners	360 (34.4)	268 (34.8)		92 (33.3)		**<0.001 ^2^**
	Service	302 (28.8)	221 (28.7)		81 (29.3)		
	Manual or unemployed	71 (6.8)	38 (4.9)		33 (12.0)		
	Retired	313 (29.9)	243 (31.6)		70 (25.4)		
Offspring	Nulliparous	284 (27.1)	191 (24.7)		93 (33.6)		**0.005 ^2^**
	Parous	765 (72.9)	581 (75.3)		184 (66.4)		
Smoke	Non-smokers	889 (84.7)	667 (86.4)		222 (80.1)		**0.013 ^2^**
	Smokers	160 (15.3)	105 (13.6)		55 (19.9)		
Alcohol consumption	Non-regular	760 (72.4)	559 (72.4)		201 (72.6)		0.961 ^2^
	Regular	289 (27.6)	213 (27.6)		76 (27.4)		
Physical activity	Non-regular	464 (44.2)	316 (40.9)		148 (53.4)		**<0.001 ^2^**
	Regular	585 (55.8)	456 (59.1)		129 (46.6)		

OPS, Online Prevention Survey; ^1^ t-test; ^2^ χ^2^ test; boldface indicates statistical significance (*p* < 0.05).

**Table 2:** Type of secondary prevention and negative predictors.

Type, Frequency and Quantity of Secondary Prevention and Negative Predictors		Group P
		*N* (%)
Type of prevention	Cardiovascular prevention	97 (12.6%)
	Oncological prevention	628 (81.3%)
	Other types of prevention	106 (13.7%)
Frequency of prevention	Only one time	48 (6.3%)
	Annual	341 (44.8%)
	Biannual	308 (40.5%)
	Every three years	64 (8.4%)
Quantity of prevention	Only one type	440 (57.7)
	Two types	190 (24.9)
	More than two types	133 (17.4)
		**Group NP** ***N* (%)**
Negative predictors	Disregard	84 (30.4%)
	Uneasiness	8 (2.9%)
	Fear of the outcome	26 (9.4%)
	Distrust in health care facilities	10 (3.6%)
	Logistic barriers	85 (30.8%)
	Health problems	22 (8.0%)
	Other reasons	22 (8.0%)
	Disinformation	6 (2.2%)
	Laziness	5 (1.8%)
	Missing data	8 (2.9%)

In “other types of prevention”, we gathered the following exams: eye exams, blood tests, ENT exams, and dental exams. Moreover, the three main prevention categories (i.e., cardiovascular, oncological, and other types) were not self-excluding to each other: for example, a participant that had indicated both cardiovascular and oncological prevention was included in both cardiovascular prevention and oncological prevention categories.

**Table 3:** Psychological characteristics.

Psychological Variables		Group P	Group NP	*p*-Value
		Mean (SD) or median (IQR)	Mean (SD) or median (IQR)	
BDI-II		**8 (4–13)**	**10 (5–16)**	**<0.001 ^1^**
PSS		14.63 (7.1)	16.82 (8.5)	**<0.001 ^2^**
GSE		29.04 (4.5)	28.35 (5.2)	**0.033 ^2^**
STAI-T	Trait anxiety	38.92 (11.2)	42.22 (12.4)	**<0.001 ^2^**
HAQ	Interference	0 (0–2)	1 (0–2.5)	**0.039 ^1^**
	Fear of death/illness	5 (3–8)	6 (3–9)	0.183 ^1^
	Worry about health	6 (4–9)	7 (3–10.5)	0.438 ^1^
	Reassurance	3 (2–4)	2 (1–4)	**0.024 ^1^**
	Total score	15 (10–22)	16 (9.5–25)	0.422 ^1^
MSPSS	Family	5.17 (1.3)	4.87 (1.5)	**0.002 ^2^**
	Friends	4.84 (1.2)	4.43 (1.4)	**<0.001 ^2^**
	Significant others	5.51 (1.2)	5.30 (1.4)	**0.016 ^2^**
	Total score	5.17 (1.1)	4.87 (1.2)	**<0.001 ^2^**
DS-14	Negative affectivity	9 (4–15)	11 (5–17)	**0.001 ^1^**
	Social inhibition	7 (3–13)	10 (3–15)	**0.003 ^1^**

BDI-II, Beck Depression Inventory-II; PSS, Perceived Stress Scale; GSE, General Self-Efficacy Scale; STAI-T, State-Trait Anxiety Inventory—Trait anxiety; HAQ, Health Anxiety Questionnaire—Total score; MSPSS, Multidimensional Scale of Perceived Social Support—Total score; DS-14, Type-D Scale-14; boldface indicates statistical significance (*p* < 0.05); ^1^ Mann–Whitney U test; ^2^ t-test.

**Table 4:** Logistic model.

Logistic Regression Predictors		OR	95%CI
Age		1.08	**1.06–1.09**
Sex	Men	0.31	**0.22–0.43**
Physical activity	Regular	1.48	**1.08–2.02**
Occupation	Retired	0.67	0.43–1.05
	Service	1.12	0.77–1.64
	Manual or unemployed	0.48	**0.29–0.79**
BDI-II		0.97	**0.96–0.99**
MSPSS total score		1.02	**1.01–1.04**

OR, Odds Ratio; CI, Confidence Interval. The “sex” reference category was “women”. The “physical activity” reference category was “no physical activity”. The “occupation” reference category was “manager or practitioners”. Boldface indicates statistical significance (*p* < 0.05). The multivariate logistic model potential predictors were age, education, gender, occupation, alcohol consumption, physical activity, smoking behavior, offspring, Beck Depression Inventory-II (BDI-II), Type-D Scale-14 (DS-14), Multidimensional Scale of Perceived Social Support—Total score (MSPSS), State-Trait Anxiety Inventory—Trait anxiety (STAI-T), Health Anxiety Questionnaire—Total score (HAQ), Perceived Stress Scale (PSS), General Self-Efficacy (GSE).
